# Transformation of the WHO Africa Region Secretariat: an exploratory study of the health policy lessons from health governance strengthening interventions for just and sustainable health systems

**DOI:** 10.3389/fpubh.2025.1616655

**Published:** 2025-07-24

**Authors:** Bernard Hope Taderera

**Affiliations:** Faculty of Health Sciences, Department of Environmental Health, University of Johannesburg, Johannesburg, South Africa

**Keywords:** transformation, WHO Africa Region, health policy, lessons, just and sustainable, health systems

## Abstract

**Introduction:**

The World Health Organization African Region (WHOAFRO) Transformation Agenda initiated in 2015 has emerged as an important health leadership strengthening initiative in pursuing the 2030 health Sustainable Development Goals (SDGs) and universal health coverage. However, there remains need for research narrating health policy lessons from implementing it with a focus on Pro-Results Values, Smart Technical Focus, Responsive Strategic Operations, and Effective Communications and Partnerships, for just and sustainable public health systems in pursuing universal health coverage.

**Aim:**

This study explored health policy lessons from the implementation of WHOAFRO Transformation from 2020 to 2023, and how this may help reinforce health systems governance on the continent.

**Methods:**

This paper is generated from a wider study commissioned by WHOAFRO. The study design used was exploratory and qualitative. Secondary data were collected from publicly available purposively selected WHO documents. Narratives were first generated from the data, followed by coding and then thematic analysis.

**Results:**

Training and mentorship helped strengthen skills levels amongst health leaders which helped contribute efficiently toward Pro-Results Values. Interventions were directed to align with continental health system leadership goals for Smart Technical Focus. Leadership structures and systems were streamlined to strengthen organizational functions for the delivery of desired outcomes to achieve Responsive Strategic Operations. Collaboration with donors and health system governance partners at all levels helped broaden the resource base, which was complemented by the adoption of digital tools and hybrid work arrangements for Effective Partnerships and Communication.

**Discussion and conclusion:**

Transforming health leadership in the Africa Region requires sustained effort to train health leaders to help strengthen health systems governance toward meeting the healthcare needs of the population. There is need for continued reinforcement of ethics values amongst health leadership to maintain a high moral standards for health workforce protection. There is also a need to sustain innovativeness in incorporating digital tools and hybrid work models into health governance for effective and efficient communication in health system leadership work. Strengthening partnerships between actors at all health governance levels helps contribute toward resource mobilization required for health system functioning for just and sustained pursuit of universal health coverage.

## 1 Introduction

Transformation of the World Health Organization (WHO) Secretariat in the African Region, is a health leadership strengthening initiative launched by the WHO Africa Regional Director in 2015, and endorsed by the Sixty-fifth session of the Regional Committee for Africa. The aim of this health governance capacity strengthening intervention is to help reinforce healthcare systems across the continent in pursuing the 2030 Sustainable Development Goals (SDGs), with a particular focus on Goal 3, and toward the realization of universal health coverage (1). The vision behind transformation is the emergence of a capacitated, adequately resourced, appropriately equipped, foresighted, results-driven, effective, efficient, transparent and accountable WHO that staff and stakeholders want, and one that is a primary leader in the preparation for and response to Public Health Emergencies of International Concern, and in delivering its mandate to strengthen country-level health systems on the continent toward meeting the healthcare needs and expectations of the population in a just and sustainable manner (2–4).

There has so far been four phases through which Transformation of the WHO Regional Office for Africa has been implemented. In the initial phase, implemented between 2015 and 2018, the focus was on creating the WHO that stakeholders want and deserve (5, 6). This was followed by a second phase, from 2018 to 2020, which focused on placing people, staff and populations in Member States at the center of change with the aim of transforming the Secretariat's organizational culture for country-level impact. In phase three (2021 to date), the aim is to consolidate the transformation through institutionalization of change (1, 3, 7). These three phases resulted in the formulation of four integrally connected focus areas within which progress in the transformation process was to be made. These four focus areas, outlined in Table 1, include Pro-Results Values, Smart Technical Focus, Responsive Strategic Operations, and Effective Communications and Partnerships (3–6).

**Table 1:** The four focus areas of the transformation agenda of the WHO Secretariat in Africa.

Pro-results values	Seeks to enhance an institutional culture characterized by values of excellence, teamwork, accountability, integrity, fairness, innovation and openness through six workstreams namely: (i) strengthening change management processes and enhancing a value-based culture; (ii) enhancing the country focus approach for greater impact; (iii) delivering quality results and value for money; (iv) promoting efficiency and accountability; (v) broadening the engagement with Member States and partners; and (vi) ensuring more effective communication of the work of the Secretariat toward improving health outcomes in the Region
Smart technical focus	Aims to ensure that the technical areas of the WHO Secretariat in the Africa Region's work are in line with regional priorities and commitments, and interventions that are based on evidence, innovations and lessons learnt from experience
Responsive strategic operations	Was adopted to help the WHO Secretariat to evolve into an organization with enabling functions that efficiently support the delivery of goods and services
Effective communication and partnerships	Has the goal to foster a more responsive and interactive organization, internally among staff members and externally with stakeholders. This has to do with enhancing effective communication internally between staff members and externally with stakeholders, build lasting relationships and strengthen transparency and trust

Source: World Health Organization (3–6).

However, whilst transforming the WHO Secretariat in Africa has made considerable progress, the outbreak of the Coronavirus Disease of 2019 (COVID-19), declared a Public Health Emergency of International Concern (PHEIC) by WHO on 30 January 2020, and a pandemic on 11 March 2020 affected implementation (3, 7). Even though the WHO Director General through the 15th meeting of the International Health Regulations (2005) (IHR) Emergency Committee on the COVID-19 pandemic held on 4 May 2023 ended the WHO PHEIC declaration, taking effect from 5 May 2023, there remains room for research exploring the health policy lessons from the implementation of WHO Transformation during public health emergencies of international concern with a particular focus on the Africa Region Secretariat (2020–2023) and how this may help in health leadership strengthening in pursuing healthcare system goals (7, 8). In this regard, this paper explores the emerging health policy lessons focusing on the four focus areas of the WHO Transformation Agenda, and how this may help reinforce health systems in pursuing Sustainable Development Goal 3, Good Health and Well-Being, to ensure healthy lives and promote wellbeing for all at all ages, and universal health coverage. For this purpose, health policy is defined as purposive course of action and interventions undertaken by health leadership with the goal to enable health systems strengthening and functioning so as to achieve desired health outcomes. Health governance in the context of this paper is defined as health systems leadership to enable the strengthening and functioning of the healthcare system, structure and processes for the achievement of health goals (3).

## 2 Methods

### 2.1 Study design

An exploratory qualitative research design was used. In this, the aim was to explore the health policy lessons which emerged in the four focus areas from the implementation of the Transformation Agenda of the WHO Africa Secretariat (9).

### 2.2 Research setting

This paper is generated from a study commissioned by the WHO Regional Office for Africa titled “The Transformation Agenda of the WHO Secretariat in the African Region 2020–2023: Reflecting on health policy lessons from COVID-19 toward reinforcing health system strengthening for achieving universal health coverage by 2030.”

### 2.3 Units of analysis and data collection

Secondary data were sourced from a publicly available dataset accessed from the Transformation Section of the WHO Regional Office for Africa website (10, 11). In this, the units of study were WHO Africa policy documents, annual reports and committee reports narrating the implementation of the Transformation Agenda by the WHO Regional Office for Africa between 2020 and 2023 (11). These policy documents were purposively selected for this study. Thereafter, a documentary search was carried out to establish the health policy lessons which emerged from implementation and how they may help reinforce health governance for the realization of healthcare system strengthening in the WHO Africa Region and beyond (9).

### 2.4 Presentation and analysis of data

Narratives of the qualitative data collected were first generated manually before being coded. Thereafter, the coded narratives were then subjected to interpretive thematic analysis. However, we established that there were quantitative figures in the dataset and we used these to support qualitative narratives (9, 10).

## 3 Results

### 3.1 Pro-results values

It was established that the Diversity, Equity and Inclusion (DEI) initiative was implemented during this period (2020–2023). DEI implementation resulted in the number of Female Team Leads, Heads of Country Offices and Directors increasing by 6.6% from 24.2% in 2020 to 30.8% at the beginning of 2022. Additionally, a second exclusive female staff cohort of the WHO Africa Pathways to Leadership Programme, an intensive 3-month programme launched toward the end of 2021 to help strengthen leadership skills for teamwork, communication and coaching, and enabling the trainees to create a leadership vision aligned with WHO or ministries of health values, outlined in Table 2, resulted in an increased percentage of female staff equipped with the requisite leadership competencies to effectively transform Africa's health from 38% in 2019 to 48% in 2022 (12). In 2023, this Pathways to Leadership for Health Transformation Programme was also extended to other regions of the WHO and health ministries. In that, a cohort of 20 senior staff from the European Region, a joint WHO Africa/Europe Cohort of 23 senior staff, and another joint Africa/Europe Cohort of 20 WHO Representatives (WRs) and staff on their roster were trained. Benin also launched its first Cohort of Directors during the same period whilst WHO Ghana devoted its fourth cohort to women thereby resulting in an all-female cohort for the leadership programme at country-level with the goal. In this, Ghana sought to enhance the leadership skills of women in the health sector and to align its interventions with the agenda of the Ministry of Health in order to allocate 60% of leadership training opportunities to women (13).

**Table 2:** Main lessons and summary of results (2020–2023).

**Lesson**	**Interventions**	**Summary of results**
**Pro-results values**		
*Reinforcing leadership helped sustain the WHO Regional Office for Africa's Transformation*	Implementation of the Diversity, Equity and Inclusion (DEI) initiative	• 6.6% increase in the number of Female Team Leads, Heads of Country Offices and Directors from 24.2% in 2020 to 30.8% at the beginning of 2022 (11)
	WHO leadership programme for member states in the Africa Region	• Launched in August 2020, with 34 women from the Republic of Congo participating in the pilot phase (5) • 180 mid-level and senior managers trained in Eight Cohorts within which 50% were women (14)
	Continued implementation of the WHO in Africa pathways to leadership for transformation programme	• Second exclusive female staff cohort resulted in an increase in the proportion of female staff trained, from 38% in 2019 to 48% in 2022 (11) • extended to other WHO Regions and ministries of health thereby resulting in training of a cohort of 20 senior staff from the European Region, a joint WHO Africa/Europe Cohort of 23 senior staff, and another joint Africa/Europe Cohort of 20 WHO Representatives (WRs) and staff on their roster in 2023 • Launch of first Cohort of Directors in Benin in 2023 • Fourth Cohort devoted to women in Ghana (12) • Percentage of female staff equipped with the requisite Africa Health Transformation Leadership Competencies increased from 38% in 2019 to 48% in 2022 (11)
	The women in leadership speaker series	• Launched in 2021 • Female staff participants from the three sessions conducted between November 2021 and April 2022 reported increased confidence levels and a growing sense of belonging (11)
	Continued implementation of the mentorship programme	• Six-month programme launched in 2020 matching mentors and mentees based on shared values (14) • First Cohort of 21 mentors and mentees paired was followed by a Second Cohort in September 2020, and then the launch of a Third Cohort in 2022, a new milestone acknowledged within WHO globally (11, 14) • An October 2020 mid-term assessment of the first cohort established, from mentors, improvements in coaching and active listening skills and an enhanced appreciation of different cultures in the Organization, while 96% of mentees reported experiencing learning and growth as a result of the mentorship relationship (5) • 70% of participants experienced increased effectiveness in their work and felt the programme was worth their time and effort (14) • Ranked as one of the best initiatives supporting staff in building their capacity based on their professional and career development needs • 300 Mentees from the WHO African Region and 140 Mentors from across all WHO major offices including WHO headquarters were successfully matched and achieved the key outcomes of this programme by 2022 (11) • Mentorship along with Team Performance incorporated into staff development and learning activities to streamline and integrate training through “a one-tool,” “one-concept” and “one-mentor strategy” • Launch of Fourth Cohort, with 99 Mentees and 37 Mentors in 2023 • Second round of 360-Degree Feedback conducted among WHO Country Representatives and Operations Officers to help consolidate awareness and a culture of feedback among managers (12)
	The Team Performance Programme	• Team Performance together with the Mentorship Programme incorporated into staff development and learning activities to streamline and integrate training through “a one-tool,” “one-concept” and “one-mentor strategy • Team Performance Feedback Sessions carried out with Eleven Regional Office units and three countries conducted to identify ways and means of consolidating change achievements (12)
*Committing to the embrace of WHO values is helping toward the upholding of the highest ethical standards in the organization*	Ombudsman and a Regional Coordinator for Prevention and Response to Sexual Exploitation, Abuse and Harassment (PRSEAH)	• Recruitment of an Ombudsman and a Regional Coordinator for PRSEAH in the first quarter of 2022 (11) • 253 training WHO staff training sessions and 1021 sessions with communities, organized by WHO Africa to help address PRSEAH (14)
	Introduction of proactive measures to help promote workplace mental health during COVID-19 and to adapt in the shift to the new ways of virtual and hybrid working	• Flexible working arrangements are now in place • Recruitment of two dedicated Staff Counselors, and a Staff Development and Learning Officer to advise and support both staff and managers in effectively navigating the new WHO way of working (11)
	Sharing of good transformation practices within WHO	• The WHO Secretariat in the Africa Region shared some of its successful experiences, including the functional review of country offices, change management, and the use of key performance indicators • Innovative corporate and management practices by the WHO Regional Office for Africa have influenced the global transformation and are being replicated across WHO • WHO Secretariat in the Africa Region provided leadership training for staff from other WHO regions (12)
*Close partnership with country offices helped fast-track country-level Transformation initiatives*	Enhancing WHO country presence by strengthening WHO-country partnership for strong health sector leadership in the Region	• Completion of the functional reviews across the 47 country offices and Regional Office clusters by 2022 • Establishment of 11 Multi-Country Assignment Teams throughout 2022 (11)
	Fast-tracking the strengthening of national public health security capacities to prepare for and respond to the region's extensive public health emergencies	• As of April 2022, all the 47 Member States had polymerase chain reaction (PCR) laboratory capacity for confirming COVID-19 • 39 Member States with the exception of Angola, Burundi, Cabo Verde, Central African Republic, Comoros, Eritrea, Liberia and South Sudan, had sequencing capacity for identifying circulating SARS-CoV-2 variants • Developing and providing access to authoritative case management guidance in 2021 • Deployment of 809 experts and training of 200,000 community health workers in risk communication across the Region • Drop in case fatality ratios by 1% and nearing the 1.4% global average • 139,703,101 children under 15 years of age in nine Member States vaccinated against potential outbreak and circulation of Vaccine-Derived Poliovirus Type 2 (cVDPV2) (11) • the independent Africa Regional Certification Commission officially declared the WHO African Region free of Indigenous Wild Poliovirus in August 2020 (5) • Vaccination of 11,000 people against Ebola in Guinea • Deployment of 40 Programme Management Officers and 33 External Relations Officers to strengthen technical assistance and empowerment of WHO Country Teams to deliver country-level impact in the 47 WHO Country Offices (11)
	Strengthening technical support provided by the multi-country assignment teams and investing in human capital and resources	• Deployed 11 multi-country assignment teams consisting of at least 95% subject matter experts whose impact is being felt on the ground • Establishment of three flagship programmes, namely promoting resilience of systems for emergencies, transforming Africa's surveillance system, and strengthening and utilizing response groups for Emergencies to help respond to emergencies • Trained seven dedicated National Emergency Response Teams in different emergency response modules and equipped them with resources and technology to respond to emergencies • Launched the Mwele Malecela Mentorship Programme for Women in Neglected Tropical Diseases to strengthen the professional growth of individuals in the field of Neglected Tropical Diseases • Initiation of the Documentation and Communication Initiative for the WHO Transformation interventions to promote accountability and learning, monitor progress and enhance knowledge management • Recording how country offices, in collaboration with partners, continued to address the HIV/AIDS epidemic (12).
**Responsive strategic operations**		
*Respond to the demand from Member States to strengthen leadership and change management during public health emergencies*	Introduction of the blended pathways to leadership for health transformation programme	• Piloted the Pathways to Leadership for Health Transformation Blended Programme in Congo, to senior ministry of health officials in Ghana, Lesotho and Niger • Over 100 ministry of health participants already applying their newly acquired skills in strengths-based leadership and systems thinking in leading health system recovery efforts and addressing health problems affecting their population
	Continued incorporation of Digital Tools into daily work	• Microsoft Teams helped WHO teams to remain connected and focused • Introduced the new Translation Management System (TMS) which resulted in efficient processing of translation requests • The WHO Secretariat in Africa supported over 350,000 field workers and more than 200,000 polio campaign workers across 16 Member States in the transition from cash to digital payments which improved cost effectiveness and reduced lag time in financial reporting (11)
	Diligence in enhancing internal accountability, demonstrating value for money and tracking the immediate gains of health interventions, the Secretariat's efforts to secure long-term agreements and broadening of the supplier base	• The generation of efficiency gains of approximately US$ 1.6 million • A strengthened WHO supply chain in the African Region (11)
	Fostering stakeholder feedback to teams in the six dimensions	• Feedback from this enabled teams to fully understand where they stand in terms of implementing change, locating transformation challenges and focusing on the most impactful actions • Twelve units and two clusters in the Regional Office and four WHO country offices (Benin, Sao Tome, Algeria, and South Sudan) are used the lessons learnt from the feedback to address collective performance challenges (12)
	Promoting mental health and fostering a healthy work environment	• WHO supported its staff and their dependants by providing counseling and psychological services and introducing collaboration tools to facilitate hybrid meetings which improved staff productivity and enabled more flexibility in working (12)
	Addressing COVID-19 pandemic-related needs by establishing clinical sites for testing and vaccination	• Improved follow-up, management and medical care for patients, and the provision of PCR testing equipment and kits • Convenient locations of the vaccination sites resulted in high levels of staff vaccination coverage which reached over 95% by 2022 • Promoting the health and wellbeing of staff and the importance of a healthy work environment in fostering mental health and productivity (12)
	Delivering quality results and value for money	• In 2020, the Secretariat introduced value-for-money performance indicators for all technical units to maximize the impact of every dollar spent (5)
	Mid-term review and the definition of clear performance metrics and goals for health interventions, and regular monitoring and evaluation of progress against these metrics	• Introduction of the Mid-term Review Tool helped facilitate internal accountability and ensure that health interventions provide value for money • Risk, compliance, administrative reviews and internal audits were conducted in 14 countries to promote a culture of transparency and accountability and ensure compliance with policies and procedures; • A follow-up of 14 internal and four external audit report recommendations of the previous year incorporated into the implementation of appropriate actions to address the identified issues and internal control gaps • Risk management briefings and training sessions for multiple and targeted budget center personnel conducted in 26 budget centers (12)
	Ensuring regulations of the COVID-19 vaccines.	• Initiation of the consultations with the African Vaccine Regulatory Forum for a joint review process in the development of COVID-19 diagnostics, vaccines and medicines (12)
**Effective partnerships and communications**		
*Focus on transforming the approach to partnerships and resource mobilization*	Scaling-up the region-wide roll-out of the digital contributor engagement management system	• More than 200 staff trained in the use of the Contributor Engagement throughout 2022 • Provided an added opportunity through which to expand the funding base, and to diversify and broaden the range of philanthropic and new donors • Over 150 non-state actors engaged and brought together which helped increase the impact of WHO interventions at country level • Strategic partnerships were forged government contributors, and partnerships were strengthened with member organizations of the Harmonization for Health in Africa mechanism through the new Framework for Better Collaboration and Greater Impact at Country Level 2022–2023 • Concerted efforts to transform external relations and resource mobilization translated into over 30% (US$ 622 million) of the allocated budget for the 2020–2021 biennium for the Region (US$ 1.7 billion) being directly mobilized by the WHO Secretariat in Africa (11)
	Engagement of key influencers to help in communicating COVID-19 interventions	• More than 90 of the over 200 videos promoting COVID-19 healthy practices got one million or more views on the WHO Secretariat Africa's social media platforms, mainly Facebook and Twitter • The WHO Secretariat Africa's social media platforms grew by 1.129 million followers compared to the annual target of 600,000 • Thirty-two newsletters narrating the main elements of the pandemic response were disseminated to ministries of health, United Nations agencies and donors with a unique open rate of nearly 40%, a 10% increase from the previous year • The African Infodemic Response Alliance formed in 2020 to counter public health misinformation grew in membership from 13 to 1,514 • The Viral Facts Initiative which emerged from this is being used as a communication tool to dispel myths, misconceptions and disseminate trustworthy COVID-19 messages • In this, the African Infodemic Response Alliance COVID-19 social content hub, produced and disseminated over 100 different pieces of digital content countering damaging public health misinformation in Africa in multiple languages, generating over 95 million views (11) • The WHO Secretariat for Africa launched a micro-website on the achievements of the Transformation Agenda, and increased its social media presence and engagement, reaching an audience of over 150 million through 29 media campaigns (12)
	Enhancing external donor relations	• Partnership was strengthened with donors and the funding base diversified to include global and continental private sector actors resulting in the mobilization of over US$ 3.5 million toward COVID 19 pandemic support to member states in 2021 (5) • The WHO Secretariat in Africa shared donor-focused communication products acknowledging 24 and publishing close to 250 communication products, reaching out to an audience of over 6 million people [13] • Recruitment of eight External Relations Officers to help provide expertise in pursuing funding opportunities (12) • In 2022, the WHO Secretariat in Africa introduced regular reporting and feedback mechanisms and organized over 60 partner briefings to foster a better understanding of, and collaboration in its work thereby resulting in the mobilization of new funding at the country level, totalling US$ 422 million • The WHO in Africa Regional Office also increased collaboration with non-State actors, signing 112 agreements worth over US$ 60 million for efficient strategy implementation • WHO Africa partnered with academic institutions to expand and preserve the Leadership for Health Transformation Programme • Increased collaboration with other United Nations agencies, engaging in 31 agreements or joint programmes (12)

Furthermore, the Women in Leadership Speaker Series launched during the same period enabled women to interact with female leaders in global development and to acquire skills to handle professional and other leadership challenges. We also noted that the Women in Leadership Speaker Series helped bring together WHO staff, regardless of their gender, and high-ranking African female leaders who worked in the health and development to engage in candid career advancement and leadership development conversations. Feedback from participants of the three sessions of this series conducted between November 2021 and April 2022 revealed improvements in female staff confidence levels and a growing sense of belonging. We also established that the Mentorship Programme launched its third cohort during the same period, a new milestone for this intervention. This programme was also acknowledged within WHO globally as one of the best initiatives that supported staff in building their capacity based on their professional and career development needs. From this, 300 Mentees from the WHO African Region and 140 Mentors from across all WHO major offices including WHO headquarters were successfully matched and achieved the key outcomes of the Mentorship Programme by 2022 (12). To further strengthen this intervention, the WHO Africa Mentorship Programme, along with the WHO in Africa Team Performance Programme were, in 2023, incorporated into staff development and learning activities through an approach that emphasized the need to streamline and integrate training through a one-tool, one-concept and one-mentor strategy. In the same year, the Mentorship Programme launched its Fourth Cohort, with 99 Mentees and 37 Mentors. To consolidate awareness and a culture of feedback among managers, a second round of 360-Degree Feedback was conducted among WHO Country Representatives and Operations Officers. Eleven Regional Office units and three countries conducted the team performance feedback sessions to identify ways and means of consolidating change achievements (13).

Transformation during the 2020–2023 period also showed the need to uphold WHO values and ethics standards. In this, the Prevention and Response to Sexual Exploitation, Abuse and Harassment (PRSEAH) was initiated to help prevent and address harassment and abuse of authority. An Ombudsman and a Regional Coordinator were recruited in the first quarter of 2022 to help reinforce the implementation of PRSEAH (12). A total of 253 training sessions with staff and 1021 sessions with communities were also carried out since 2021, resulting in more awareness and meaningful conversations on this challenge and incorporation into operational processes for staff wellbeing (5). To further protect workers, WHO introduced workplace mental health initiatives to protect the mental wellbeing of staff members in order to sustain and improve their productivity during COVID-19 and cope with the shift to virtual and hybrid working. Two Staff Counselors and a Staff Development and Learning Officer were recruited to help support both staff and managers in their mental welfare (12). Good transformation practices were also shared within WHO through meetings and communication, and Cross-Functional Teams. This brought together staff from different departments and locations to share perspectives, skills and experiences. Furthermore, we established that these practices influenced global transformation and are being replicated across WHO, while the WHO Secretariat in the Africa Region has gone on to provide leadership training for staff from other WHO regions (5).

### 3.2 Smart technical focus

It was established that the COVID-19 pandemic showed the importance of close partnership between the WHO Secretariat and its country offices to fast-track country-level transformation initiatives and to enhance WHO country presence and health sector leadership. In pursuing this, progress was made through the completion of functional reviews across the 47 country offices and Regional Office clusters as outlined in Table 2. Efforts to bring timely, high-quality technical support closer to countries culminated in the establishment of 11 Multi-Country Assignment Teams who brought specialized technical experts to work closely with the country offices to scale up technical support across eight critical health areas during this period. The WHO Secretariat in the Africa Region also used the COVID-19 pandemic data and experiences of 2019–2023 to fast-track the strengthening of national public health security capacities required for the preparation for and response to the region's extensive public health emergencies (12). On this, it was established that by April 2022, all the 47 Member States had Polymerase Chain Reaction (PCR) laboratory capacity for confirming COVID-19 whilst 39 Member States with the exception of Angola, Burundi, Cape Verde, Central African Republic, Comoros, Eritrea, Liberia and South Sudan, had sequencing capacity for identifying circulating SARS-CoV-2 variants. In addition, the Secretariat strengthened COVID-19 preparedness, readiness and response capacities, including developing and providing access to authoritative case management guidance, deployment of 809 experts and training of 200,000 community health workers in risk communication across the Region. Consequently, this resulted in a drop in case fatality ratios by one percentage point by the end of 2021, a figure which was closer to the 1.4% global average (12).

Significant progress was also made from interventions to end all forms of Polio in the WHO Africa Region with over 139,703,101 children under 15 years of age in nine Member States vaccinated against potential outbreak and circulation of Vaccine-Derived Poliovirus Type 2 (cVDPV2). In August 2020, the independent Africa Regional Certification Commission (ARCC) officially declared the WHO African Region free of indigenous wild poliovirus. This achievement was attributed to the leadership of Member States, effective collaboration among polio partners, and the adoption of innovative approaches, such as the use of Geographic Information Systems (GIS) to strengthen surveillance (5, 12). The WHO Regional Office for Africa also responded to the Ebola outbreak in Guinea, and supported the vaccination of 11,000 people which helped curtail the spread of the disease. In addition, the WHO Secretariat deployed 40 Programme Management Officers (PMOs) and 33 External Relations Officers (EXOs) to 47 WHO Country Offices which helped strengthen technical assistance and empowered WHO Country Teams to deliver country-level impact (12).

The WHO Regional Office for Africa also established three flagship programmes, namely Promoting Resilience of Systems for Emergencies (PROSE), Transforming Africa's Surveillance System (TASS), and Strengthening and Utilizing Response Groups for Emergencies (SURGE), to help respond to emergencies as part of its Smart Technical Focus. During 2022, seven dedicated National Emergency Response Teams were trained on different emergency response modules and equipped with the necessary response resources and technology. This enabled them to respond in a timely and effective manner to the emergency outbreaks of Rift Valley Fever in Mauritania, Polio in Botswana, Cholera in Niger and Meningitis in Togo. In addition, the WHO Secretariat in the Africa Region conducted risk assessments and contingency planning exercises in 19 countries in anticipation of and preparation for potential emergencies. Furthermore, the WHO Regional Office for Africa also launched the Mwele Malecela Mentorship Programme for Women in Neglected Tropical Diseases in October 2022, to strengthen the professional growth of individuals in the field of Neglected Tropical Diseases (NTDs). This programme provided mentees with the opportunity to work with experienced mentors and receive guidance, advice and support in their careers for skills development, exposure to new ideas and perspectives, and relationship building within the NTD community. The overall aim was to provide them with the resources and support they required to succeed in their careers, become influential leaders and make a positive impact toward the prevention and control of NTDs in Africa. The WHO Secretariat in the Africa Region also recorded how country offices, in collaboration with partners continued to address the HIV/AIDS epidemic in West and Central Africa, and monitored Antiretroviral Treatment (ART) coverage in the Region throughout the time between 2020 and 2023. From this, it was established that although several countries in West and Central Africa achieved 90% coverage for ART, progress in the control of the HIV/AIDS epidemic was hindered by insufficient political commitment, health system weaknesses and serious health challenges such as the outbreak of COVID-19 and Ebola (13).

### 3.3 Responsive strategic operations

During the 2020-23 period of the COVID-19 outbreak, the WHO Regional Office for Africa responded to the growing demand from Member States to strengthen leadership and change management competencies by expanding the Pathways to Leadership for Health Transformation Blended Programme, outlined in Table 2. This blended programme was piloted in Congo, and to senior ministry of health officials in Ghana, Lesotho and Niger. From this, 100 health ministry participants were trained and applied their newly acquired skills in strengths-based leadership and systems thinking to lead health system recovery efforts and address some of the core health challenges which affected their population. In addition, digital tools such as Microsoft Teams helped facilitate organizational agility, remote work and enhanced efficiency by helping WHO teams to remain connected and focused during the COVID-19 lockdown. This was complemented by the introduction of a new Translation Management System (TMS) which led to efficiencies in the processing of translation requests. Furthermore, digital solutions were rapidly scaled up across the Secretariat's operations thereby resulting in over 350,000 field workers and more than 200,000 polio campaign workers across 16 Member States moving from cash to digital payments. In turn, this resulted in improved cost effectiveness, reduced lag time in financial reporting and reduced inequities through financial inclusion of the rural poor. In addition, diligence in enhancing internal accountability, demonstrating value for money and tracking the immediate gains of health interventions along with the Secretariat's efforts to secure long-term agreements and broaden the supplier base which resulted in the generation of approximately US$ 1.6 million and contributed to a strengthened WHO supply chain in the African Region by the end of 2021 (12).

To further enhance internal accountability so as to ensure value for money, the WHO Africa Secretariat introduced the Mid-term Review Tool, defined performance metrics and goals for health interventions, and regular monitoring and evaluation of progress against these metrics. In addition, the Secretariat's robust financial management systems and processes, including budgeting, forecasting and reporting, helped ensure effective resource allocation and use. In this, risk, compliance, administrative reviews and internal audits were conducted in 14 countries to promote a culture of transparency and accountability and ensure compliance with policies and procedures. Furthermore, a follow-up of the 14 internal and four external audit report recommendations of the previous year corroborated the implementation of appropriate actions to address the identified issues and internal control gaps. Risk management briefings and training sessions for multiple and targeted budget center personnel to strengthen and create a regional risk culture complemented the assurance activities conducted in 26 budget centers (5, 13).

The WHO Regional Office for Africa also fostered stakeholder feedback to teams in six dimensions: WHO values; effectiveness; quality; cost consciousness; agility and change management; and collaboration, all as part of the efforts to effectively consolidate the change initiated under the Transformation Agenda. The objective was to enhance team effectiveness by highlighting strengths and growth areas, improving communication through constructive comments and increasing motivation and engagement. The feedback from this enabled teams to fully understand where they stood in terms of implementing change, locating transformation challenges and focusing on the most impactful actions. Twelve units and two clusters in the Regional Office and four WHO country offices (Benin, Sao Tome, Algeria, and South Sudan) used lessons learnt from the feedback to tackle collective performance challenges. To address COVID-19 pandemic-related needs amongst staff, the WHO Regional Office for Africa apart from promoting mental health, it fostered a healthy work environment by supporting its staff and their dependants through providing counseling and psychological services, and through the establishment of clinical sites for testing and vaccination against the disease. The convenient locations of the vaccination sites resulted in high levels of staff vaccination coverage, reaching over 95% by 2022. Collaboration tools to facilitate hybrid meetings were also introduced to help cope with the COVID-19 lockdown. All this helped improve staff productivity and flexibility in work arrangements. In addition, the introduction of COVID-19 vaccines in the WHO Africa Region resulted in the need for improvements in vaccine regulatory capabilities and capacities of Member States. The Secretariat initiated consultations with the African Vaccine Regulatory Forum (AVAREF) comprising of national regulatory authorities of all Member States. As a result, AVAREF endorsed an emergency joint review process and shortened timeline to accelerate the development of COVID-19 diagnostics, vaccines and medicines (13).

### 3.4 Effective partnerships and communications

It was established that transforming the approach to partnerships and resource mobilization focused on scaling-up the region-wide roll-out of the Digital Contributor Engagement Management System (DCEMS), outlined in Table 2. This system provided an added opportunity through which to access pipeline financing information, leverage different funding sources and investments and diversify the WHO funding base, thereby contributing to an increase in diversified funding from a broad range of philanthropic and new donors. More than 200 staff were trained in the use of the Contributor Engagement throughout 2022 (12). The WHO Secretariat in the Africa Region strengthened partnerships for the COVID-19 response. These included traditional partners, and governments of countries such as Belgium, Canada, Denmark, France, Germany, Ireland, Japan, Norway, Switzerland, the United Kingdom of Great Britain and Northern Ireland and the United States of America, and the Bill and Melinda Gates Foundation, the European Commission, Rotary International, the African Development Bank and the World Bank. WHO also worked closely with the Africa Centers for Disease Control and Prevention (Africa CDC), through the Africa Taskforce for Coronavirus (AFTCOR), the African Vaccine Delivery Alliance (AVDA) and the African COVID-19 Vaccine Readiness and Deployment Taskforce (ACREDT), to support coordination of the pandemic response (5, 12–14).

Partnerships were also strengthened with member organizations of the Harmonization for Health in Africa (HHA) mechanism through the new Framework for Better Collaboration and Greater Impact at Country Level 2022–2023. Furthermore, the HHA mechanism undertook a mapping exercise in 39 countries to assess the effectiveness of partner coordination and collaboration at country level, address coordination and harmonization challenges and define workable solutions for COVID-19 response. These partner transformation efforts resulted in the mobilization of over 30% (US$ 622 million) of the allocated budget for the 2020–2021 biennium for the Region (US$ 1.7 billion) directly by the WHO Secretariat in the Africa Region (12). Partnerships also resulted in the mobilization of over US$ 3.5 million toward COVID 19 pandemic support to Member States in 2021. In addition, partnerships and resource mobilization capacities of the Secretariat resulted in US$ 331.8 million mobilized for the WHO pandemic response in the African Region. This represented 72.8% of the overall budget of US$ 455.9 million in the same year. The WHO in Africa Regional Office also increased collaboration with non-State actors, signing 112 agreements worth over US$ 60 million for efficient strategy implementation. Partnerships were forged with civil society organizations and NGOs to reach the most vulnerable and remote populations (12, 15).

To help ensure effective communication of the WHO goal toward improved health outcomes since the onset of the pandemic, over 50 virtual press conferences and more than 450 media interviews involving 270 global media outlets and over 70 regional and national media organizations were delivered by the WHO Secretariat for the Africa Region. This enabled the sharing of life saving information, enhanced stakeholder engagement and demonstrated the WHO Africa Region Secretariat's health leadership. The WHO Regional Office for Africa also experienced a significant increase in followers across its social media platforms throughout the pandemic with Facebook followers increasing by 1.5 million. Visitors to the WHO Regional Office for Africa's secretariat website also increased by 283% from 1.2 million in 2019 to 4.6 million visitors in 2020. Cross-cutting internal communications on the Transformation Agenda and change management efforts also increased through the monthly “Change Management Highlights,” internal newsletters and the WHO Africa Regional Director's Townhall Meetings (5). The WHO Secretariat in Africa Region also advocated for the addressing of pressing issues such as COVID-19 vaccine equity by targeting a range of key influencers with different communication products. It also promoted healthy COVID-19 practices such as wearing of face masks, and proper hand washing, and disseminated over 200 videos. More than 90 of these videos received at least one million views on the WHO Regional Office for Africa's social media platforms, mainly Facebook and Twitter. These platforms grew by 1.129 million followers compared to the annual target of 600,000. Thirty-two newsletters showcasing key elements of the pandemic response were disseminated to ministries of health, United Nations agencies and donors with a unique open rate of nearly 40%, representing a 10% increase from the previous year. In a bid to fight COVID-19 misinformation and complement public health awareness and community engagement in the WHO Africa Region, the African Infodemic Response Alliance (AIRA) was launched by the WHO Regional Office for Africa in 2020. AIRA immediately grew in membership from 13 to 1,514. The Viral Facts Initiative which emerged from this was used as a communication tool to dispel myths, misconceptions and disseminate trustworthy COVID-19 messages (12). In this, the AIRA COVID-19 social content hub produced and disseminated over 100 different pieces of digital content countering damaging public health misinformation in Africa in multiple languages, and in the process generated over 95 million views. In addition, the WHO Secretariat for the Africa Region bolstered its external communication through a significant improvement of its online presence and brand image by revamping its website with new theme-focused pages to diversify content. This resulted in an increase in the average time spent on the website by 27%. The WHO Regional Office for Africa also launched a micro-website on the achievements of the Transformation Agenda, and increased its social media presence and engagement, which also enabled it to reach an audience of over 150 million through 29 media campaigns and better connection with its stakeholders and partners (12, 13).

To enhance accountability and transparency in its communication with donor partner, the WHO Secretariat in the Africa Region shared donor-focused communication products across various external platforms, including social media and the WHO Regional Office for the Africa Region website, in 2022. A total of 24 donors were acknowledged, and close to 250 communication products were published, including human interest stories, press releases and videos, as well as posts on Facebook and Twitter. These efforts reached a broad audience of over 6 million people, including donors and partners, the media and the general public. In addition, WHO Secretariat for the Africa Region recruited eight External Relations Officers, who leveraged their expertise to identify funding opportunities and developed proposals aligned with the needs of Member States. The increased focus on strengthening accountability and reinforcing trust with donor partners helped to further the WHO Regional Office for Africa's mission toward improved health outcomes for the continent during this period, 2020–2023. In 2022, the WHO Secretariat in Africa introduced a regular reporting and feedback mechanisms and organized over 60 partner briefings to foster a better understanding of its work, and collaborations. This resulted in the mobilization of new funding which amounted to US$422 at the country levels. To sustain leadership development among managers in health ministries and to expand and sustain the Leadership for Health Transformation Programme, the WHO Secretariat for the Africa Region partnered with academic institutions which included the Ashesi University in Ghana and the University of Pretoria in South Africa. Furthermore, the Secretariat increased collaboration with other United Nations agencies by entering into 31 agreements, joint programmes for synergistic actions and leveraging each agency's comparative advantage toward improving health outcomes in the region (13).

## 4 Discussion

The implementation of the Transformation of the WHO Secretariat in the Africa Region during the 2020–2023 period provides important lessons which may help reinforce the pursuit of the goals of Pro-Results Values, Smart Technical Focus, Responsive Strategic Operations, and Effective Partnerships and Communications (8). Health governance transformation is central to improving health system performance, in preparing and responding to public health emergencies, and in pursuing SDG 3, and universal health coverage in the Africa Region and beyond (16, 17). The first theme emerging from in discussion is that achieving Pro-Results Values of the WHO Transformation in the Africa Region requires a well-trained healthcare workforce, to lead the strengthening of country-level health systems so as to ensure the delivery of healthcare services to the population (18). In this regard, health leadership transformation equips health system leaders with knowledge and skills in organizational, team and personal management, enhance strategic and analytical capacity, and help them gain greater understanding of the complex issues encountered in governing health systems at all levels (7). However, studies on this area also emphasize the importance of training relevant to purpose, based on an understanding that there is a chain that links effective learning to high-quality services and thus to improved health. In this regard, it is important to continually and systematically examine the training of the WHO Secretariat in Africa to help ensure that it remains fit-for-purpose (18).

In addition, the study of WHO Africa Transformation (2020–2023) shows the importance of complementing basic training with mentorship programmes. Mentorship helps bridge the gap between the knowledge and skills acquired through training and practice in health leadership. However, there is need to ensure that mentorship programmes and mentoring expectations are clear and understood by both mentees and mentors. This can be achieved through discussions, learning contracts outlining mentorship outcomes and individualized development plans. Another challenge involves the establishment and maintenance of rapport between the mentors and mentees. This can be addressed through increased frequency of virtual meetings during public health emergencies of international concern. Virtual training and mentorship may however be affected by logistical challenges which may include unreliable, and often unavailable electricity supply, and unstable internet connections in some parts of Africa, and other resource limited settings around the world. One way to get around these challenges is to provide access to downloadable presentations and modules that can be viewed asynchronously, while simultaneously participating verbally through low bandwidth applications, such as WhatsApp. Internet-based training may also mitigate challenges such as cost, delay and/or inability to obtain visas to travel abroad for training, which can be amplified by the closure of country borders and embassies granting travel visas during the outbreak of diseases constituting public health emergencies of international concern. Another challenge that can be encountered in mentorship programmes is meeting the needs of trainees with different levels of technical capacity at the outset and with varied competency needs, while at the same time ensuring a diversity of trainee backgrounds, sustainability of training, and harmonization of the trainee's research and practice activities with the needs and priorities of the country where they work. This challenge can be addressed through involving local mentors in the recruitment and selection of trainees, mentoring activities and management of the program (19). Finally, training for Pro-Results Values needs to mainstream gender by reinforcing diversity, equity and inclusion with a particular focus to increase the number of women receiving training in health governance to help and ensure that more women occupy higher-level healthcare leadership positions at the WHO Regional Office in Africa, and in country offices (7). This aligns with Resolution WHA74.14 titled, Protecting, safeguarding and investing in the health and care workforce adopted at the Seventy-third World Health Assembly (WHA) of 2021, prescribing the creation of opportunities for women in the health workforce, that support their full and meaningful participation and representation, including in senior leadership and decision-making roles (20).

Our second theme is that WHO Transformation, particularly in the Africa region, needs to sustain the instilling of ethics values and standards into health leadership to help maintain a high moral and ethical standard of work for health workforce protection in line with Resolution WHA74.14 of 2021 for Pro-Results Values. Resolution WHA74.14 of 2021 calls for the safeguarding and protection healthcare workers through equitable distribution and access to PPE, therapeutics, vaccines, diagnostics, effective infection prevention, control and occupational safety and health measures within a safe and enabling work environment that is free from racial and all other forms of discrimination. In addition, this resolution also called for the prevention of violence, discrimination and harassment, including sexual harassment against healthcare workers, the majority of whom (almost 70%) are women (20). This is also in line with Resolution WHA74.14 of 2021 prescribing member states to take measures for their protection in the workspace. The implementation of the Transformation Agenda by the WHO Secretariat in the Africa Region shows the importance of creating and promoting a healthy work environment through workplace interventions which include the establishment of clinical sites providing workplace health initiatives and equipped with specialist healthcare professionals to provide counseling and psychosocial services for mental wellbeing, and testing, vaccination and if possible treatment or referral to health facilities (2, 21, 22). Furthermore, protection against discrimination, racism, tribalism, harassment, sexual exploitation and violence can be reinforced through awareness creation and meaningful conversations about the challenge amongst staff within the WHO Africa Secretariat and with stakeholders at regional, country and community levels to cultivate culture-specific, action-oriented and results-focused solutions for harmony. These can then be transformed into ethics standards, operating guidelines, protocols and regulations, for implementation in the workplace at WHO Secretariat level, in country offices, and health establishments to help protect all healthcare workers (20–22).

Theme three is that transformation of the WHO Secretariat needs to be proactive in adopting and sustaining digital tools, and in applying need-based hybrid work arrangements into health governance for Responsive Strategic Operations. The implementation of the Transformation Agenda by the WHO Regional Office for Africa between 2020 and 2023 shows that adoption of computer-based communication software such as Microsoft Teams, along with assistive Translation Software Systems, facilitate the continuation of normal day to day office communication through a digital platform for virtual meetings, broadcast of live video streams, and quick sharing of data and information during the outbreak of public health diseases of international concern which may have resulted in lockdowns, necessitating members of the Secretariat to working either in isolation from home or through a hybrid work model for productivity (12, 13). Studies suggest that Microsoft Teams has been a radical approach to digital implementation in healthcare which has so far proved effective in delivering a cost effective and rapid communication tool during the outbreak of global pandemics of public health concern (23).

Furthermore, digital tools also help facilitate connection and presence of the WHO Regional Office for Africa in society and communication with donor, partners and the world. For example, websites and social media accounts are not only information platforms but also a means through which to maintain connection with the society through followers, subscribers and interaction, through for example live virtual discussions on Facebook and Spaces on X (Twitter) for WHO presence in society. Social media platforms complement mainstream media platforms for wider delivery of information through press conferences (virtual or in-person), media interviews, and healthcare programme specific interventions. In turn this effective communication also helps complement interventions such as the Viral Facts Initiative in countering misinformation which may under otherwise undermine effective implementation of donor-focused communication products and health interventions, such as COVID-19 vaccines (12). Digital tools also help facilitate the generation of accurate, reliable, complete, consistent and timely health data to help inform effective planning and resource management in the WHO Transformation process. For instance, the transition to digital payments to healthcare workers helping the WHO Regional Office for Africa to pay community-level healthcare workers implementing interventions in different locations in the region in a timely, cost effective but also a transparent and accountable manner which in turn is also making it easy to track gains and value for money from the healthcare initiatives being implemented in the region, which in turn also helps in audits, reviews, monitoring and evaluation, and makes it easy to provide stakeholders feedback to help maintain and expand the donor base. Mobile money technology can also be used to generate heathcare data such as for monitoring and evaluation from Community Health Workers which could be integrated with Health Information Systems (HIS) within countries for improved efficiency (12, 13).

Our last theme is that transforming health governance needs to maintain and reinforce the principal-agent relationship and expand partnerships with society, governments and donor community for resource sharing and mobilization to strengthen Smart Technical Focus, and for Effective Partnerships and Communication. The principal-agent relationship between the WHO Secretariat in the African Region and in-country offices, including ministries of health, their partner ministries within countries and health governance structures therein down to the individual level within households level need to be maintained and reinforced. One main way through which to achieve this is through maintaining WHO presence for the provision of timely, responsive and relevant technical support to all health system leadership levels and ensuring community involvement and inclusion through wide decision space for responsiveness and acceptability of health interventions (12, 24). On this, a qualitative study in Kenya revealed that Community Health Committees (CHCs) are a mechanism through which communities can voluntarily participate in making decisions and providing oversight of the delivery of local healthcare services. This study however noted that for CHCs to succeed, health leadership needs to implement policies that promote wider decision space for community participation (25). This can be further reinforced by expanding collaborations with the community, governments and partnerships for resource mobilization and sharing. Partnerships with the society helps to mitigate health workforce shortages, a key health system challenge in health systems in the Africa Region. For example, a cross-sectional study carried out in Dhaka, Bangladesh, showed that household testing for SARS-CoV-2 detection by community health workers in low-resource settings is an inexpensive approach that can increase testing capacity, accessibility and the effectiveness of control measures through immediately actionable results (26). In addition, a qualitative study of Epworth, Zimbabwe reported that, despite resource constraints, community health volunteers remain an important resource through which to help complement health professionals in resource limited peri-urban areas (27). Resource constraints in transforming health systems can be mitigated by engaging and collaborations between governments and the donor community. On this, Development Assistance for Health (DAH) remains an important mechanism for funding and technical support from bilateral donors, centrally funded mechanisms, and philanthropic foundations for implementing health programmes in to lower-income countries. However, the donor-recipient dichotomy may at times create power imbalances which may result in the misalignment of priorities, short-term and often complex funding structures and processes, and a fly-in fly-out approach which can undermine aid effectiveness, WHO capacity strengthening efforts and sustainability. Call for reforming development assistance are not new to the global health community and have resulted in the formulation of aid effectiveness initiatives which include the International Health Partnership plus (2007), the Accra Agenda for Action (2008), the Busan Partnership for Effective Cooperation (2011), the Global Partnership for Effective Development Cooperation (2011), the Addis Ababa Action Agenda (2015), the Universal Health Coverage 2030 Global Compact (2017), and the 2030 Agenda for Sustainable Development which also underscores the need for a better aid model. Despite these global efforts, there has been little progress toward a more sustainable and effective model for delivering development assistance in the health sector. In this regard, the Transformation Agenda of the WHO Secretariat in the Africa Region presents an opportunity to reinforce ongoing efforts to address this challenge, toward a sustainable and effective model through which development assistance for health can be delivered in pursuing systematic and outcome-oriented health governance for strengthening of health systems in the preparation for and response to public health emergencies of international concern, and programme specific healthcare interventions for the achievement of universal health coverage and Goal 3 of the Sustainable Development Agenda in particular (28).

## 5 Conclusions

Implementing the Transformation of the WHO with a particular focus on the Secretariat in the Africa Region requires sustained effort to train healthcare personnel to lead health system reform in the region. In addition, the enforcement of standard operating norms and ethics values need to be sustained so as to protect the health workforce from violence, discrimination, harassment and harm. To complement this, there is need to sustain digital tools and hybrid work arrangements into the health leadership transformation process. In addition, it is also important to strengthen the role of women in leadership and mentoring in the region so as to help enhance and strengthen women leadership, participation and representation in decision making to help strengthen the caregiver role often played by women within families and society, and for responsive health policy interventions which help contribute toward meeting the healthcare needs of women, the family and the community. This can be further reinforced by maintaining in-country and in-community WHO presence for technical assistance, and collaboration with communities, governments and development assistance for health partners for resource mobilization and sharing to capacitate the WHO Secretariat for the realization of the health system strengthening goal of good health, equity, responsiveness in line with SDG 3, and for sustainability in pursuing universal health coverage.

## Data Availability

The original contributions presented in the study are included in the article/supplementary material. Further inquiries can be directed to the corresponding author.
